# Genomic Prediction of Autotetraploids; Influence of Relationship Matrices, Allele Dosage, and Continuous Genotyping Calls in Phenotype Prediction

**DOI:** 10.1534/g3.119.400059

**Published:** 2019-02-19

**Authors:** Ivone de Bem Oliveira, Marcio F. R. Resende, Luis Felipe V. Ferrão, Rodrigo R. Amadeu, Jeffrey B. Endelman, Matias Kirst, Alexandre S. G. Coelho, Patricio R. Munoz

**Affiliations:** * Blueberry Breeding and Genomics Lab; ‡ Sweet Corn Genomics and Breeding, Horticultural Sciences Department, University of Florida, Gainesville, FL 32611; † Plant Genetics and Genomics Lab, Agronomy College, Federal University of Goias, GO, Brazil, 74690-900; § Department of Horticulture, University of Wisconsin, Madison, WI 53706; ** Forest Genomics Lab, School of Forestry Resources and Conservation, University of Florida, Gainesville, FL 32610

**Keywords:** Autopolyploid, Allelic dosage, Genomic Selection, Relationship Matrices, *Vaccinium*, blueberry, Shared data, Genomic Prediction, GenPred, Shared Data Resources

## Abstract

Estimation of allele dosage, using genomic data, in autopolyploids is challenging and current methods often result in the misclassification of genotypes. Some progress has been made when using SNP arrays, but the major challenge is when using next generation sequencing data. Here we compare the use of read depth as continuous parameterization with ploidy parameterizations in the context of genomic selection (GS). Additionally, different sources of information to build relationship matrices were compared. A real breeding population of the autotetraploid species blueberry (*Vaccinium corybosum*), composed of 1,847 individuals was phenotyped for eight yield and fruit quality traits over two years. Continuous genotypic based models performed as well as the best models. This approach also reduces the computational time and avoids problems associated with misclassification of genotypic classes when assigning dosage in polyploid species. This approach could be very valuable for species with higher ploidy levels or for emerging crops where ploidy is not well understood. To our knowledge, this work constitutes the first study of genomic selection in blueberry. Accuracies are encouraging for application of GS for blueberry breeding. GS could reduce the time for cultivar release by three years, increasing the genetic gain per cycle by 86% on average when compared to phenotypic selection, and 32% when compared with pedigree-based selection. Finally, the genotypic and phenotypic data used in this study are made available for comparative analysis of dosage calling and genomic selection prediction models in the context of autopolyploids.

Polyploidy events are not an exception in plants, as about 70% of Angiosperms and 95% of Pteridophytes underwent at least one polyploidization event (Soltis and Soltis 1999). Polyploids are normally grouped into two categories, autopolyploids and allopolyploids, but intermediate forms are also possible, such as segmental allopolyploids (Spoelhof *et al.* 2017). Thresholds for polyploid classification have been controversial, but following the general taxonomic definition, autopolyploids arise from within-species whole genome duplication, and allopolyploids arise from whole genome duplication prior to or after an inter-specific hybridization event (Soltis *et al.* 2007).

Because speciation via ploidy increase can generate new phenotypic variability, this phenomenon is considered a powerful evolutionary source (Hieter and Griffiths 1999; Soltis *et al.* 2016). Despite the important role of polyploidization in plant evolution, its effects on inheritance of many agronomic traits and population genetics are still poorly understood when compared with diploid species (Dufresne *et al.* 2014). This especially holds true for autopolyploids. Examples of the complex nature of autopolyploid genetics are the presence of genotypes with higher allele dosage than diploids, larger number of genotypic classes, possibility of multivalent pairing, and poor knowledge of chromosome behavior during meiosis (Slater *et al.* 2014; Dufresne *et al.* 2014; Mollinari and Serang 2015).

The advent of high-throughput genotyping methods, associated with the development of genetic and statistical analysis tools, has generated significant genetic gains for diploid species (Desta and Ortiz 2014). However, the application of genomic information to polyploid crops remains a challenge (Comai 2005; Grandke *et al.* 2016). Although the theory for the computation of average genetic effect assuming arbitrary ploidy have been published by Kempthorne (1957), most of the methods for analysis and interpretation of genetic data in polyploids have only recently been described (see review in Bourke *et al.* 2018; Kerr *et al.* 2012; Endelman *et al.* 2018), and most of them have not yet been fully investigated for different species, especially for new breeding approaches, such as genomic selection.

Genomic selection (GS) is a method to increase the efficiency and accelerate the selection process in breeding programs. GS is used to capture the simultaneous effects of molecular markers distributed across the genome, based in the premise that the linkage disequilibrium between causal polymorphisms and markers allow phenotype prediction based on genotypic values (Meuwissen *et al.* 2001; Zhang *et al.* 2011; Daetwyler *et al.* 2013; de los Campos *et al.* 2013). Promising results have been reported in GS studies addressing polyploids (*e.g.*, Gouy *et al.* 2013; Annicchiarico *et al.* 2015; Ashraf *et al.* 2016), however simplified assumptions were mostly considered, in other words diploid genetic models were used to circumvent the complexity involved in accurately defining allelic dosage (*i.e.*, the number of copies of each allele at a given polymorphic locus). Besides the existence of methods that allow accounting for ploidy effects (Kerr *et al.* 2012; Endelman *et al.* 2018), only a few studies have inserted this factor in the analyses (*e.g.*, Slater *et al.* 2016; Sverrisdóttir *et al.* 2017; Nyine *et al.* 2018). In addition, these methodologies were not yet extensively compared, a point that is addressed in this article.

Polyploidy can affect phenotypes through allelic dosage (additive effect of multiple copies of the same alleles), or by creating more complex interactions between loci or alleles, such as dominance or epistasis (Osborn *et al.* 2003). Thus, the inclusion of allelic dosage information may improve GS results (*e.g.*, increase of accuracy) by creating a more realistic representation of the effects of each genotypic class. Although the evidence of dosage effects in the expression of important economic traits exists (Guo *et al.* 1996; Birchler *et al.* 2001; Adams *et al.* 2003; Osborn *et al.* 2003), few studies linking dosage effects to phenotype prediction have been reported in autopolyploid species (*e.g.*; Slater *et al.* 2016; Sverrisdóttir *et al.* 2017; Nyine *et al.* 2018; Endelman *et al.* 2018). Genotype classification is one of the major challenges for polyploids. Studies about genotyping calling evaluation for autopolyploids with next generation sequencing (NGS) data showed that none of the existing methods performs properly (Grandke *et al.* 2016), unless high sequencing coverage (60-80*x*) is used (Uitdewilligen *et al.* 2013).

Here we compare a novel approach to GS in the context of autopolyploid, using *Vaccinium corymbosum* (southern highbush blueberry, SHB) as a model. The cultivated SHB is an autotetraploid, presenting 2n = 4X = 48 chromosomes (Lyrene 2002). Inbreeding depression is strong in SHB and population improvements have been achieved by long-term recurrent phenotypic selection alongside with long testing phase and slow genetic gain per generation (Lyrene 2008). Our goal was to investigate and compare the influence of different sources of information and ploidy parameterizations used to build relationship matrices on phenotype prediction, and thus the potential of GS in blueberry breeding.

## Material and Methods

### Population and phenotyping

The population used in this study encompasses one cycle of the University of Florida blueberry breeding program’s recurrent selection, comprising 1,847 SHB unique individuals. This population was originated from 124 biparental controlled crosses, from 146 parents that presented superior phenotypic performance (cultivars and advanced stage of breeding). Phenotypic data of eight yield and fruit quality-related traits were collected during two production seasons (2014 and 2015), when the plants were 2.5 and 3.5 years of age at the University of Florida Plant Science Research and Education Unit in Citra (29°24’42.01” N -82°06’36.00” W, Florida, USA). Yield (rated using a 1-5 scale), weight (g), firmness (g mm^-1^ of compression force), scar diameter (mm), fruit diameter (mm), flower bud density (reported as buds per 20 cm of shoot), soluble solids content (^o^Brix), and pH were evaluated. The last three traits were phenotyped only in one year – soluble solids content and pH were phenotyped in 2014 and flower buds in 2015.

Five berries (fully mature and presenting picking quality) were randomly sampled to compose the measurement of fruit traits for each individual. Fruit weight was measured using an analytical scale (CP2202S, Sartorious Corp., Bohemia, NY). The FirmTech II firmness tester (BioWorks Inc., Wamego, KS) was used to measure fruit diameter and firmness. The scar diameter was obtained by image analysis of the fruits using FIJI software (Schindelin *et al.* 2012). The number of flower buds was counted in the main cane upright shoot, in the top 20 cm. A digital pocket refractometer (Atago, U.S.A., Inc., Bellevue, WA) was used to obtain soluble solids measures from 300μl of berry juice. The pH was measured using a glass pH electrode (Mettler-Toldeo, Inc., Schwerzenbach, Switzerland). More details are provided by Amadeu *et al.* (2016), Cellon *et al.* (2018), and Ferrão *et al.* (2018).

### Genotyping

Genomic DNA was extracted and genotyped using sequence capture by Rapid Genomics (Gainesville, FL). Polymorphisms were genotyped in genomic regions captured by 31,063 120-mer biotinylated probes, designed based on the 2013 blueberry draft genome sequence (Bian *et al.* 2014; Gupta *et al.* 2015). Sequencing was performed in the Illumina HiSeq2000 platform using 100 cycle paired-end sequencing. After trimming (quality score of 20), demultiplexing, and removing barcodes, reads were aligned to the draft genome using Mosaik v.2.2.3 (Lee *et al.* 2014). Genotypes were called using FreeBayes v.1.0.1 (Garrison and Marth 2012) considering the diploid and tetraploid options. Single-nucleotide polymorphisms (SNPs) were filtered considering i) minimum sequencing depth of 40 (average depth for the population); ii) minimum SNP phred quality score (QUAL) of 10; iii) only biallelic markers; iv) maximum population missing data of 0.5; and v) minor population allele frequency of 0.05. After filtering a total of 85,973 SNP were used in the GS analysis (the average sequencing-depth per sample was 73X). Further information regarding population composition and genotyping approach were described in Ferrão *et al.* (2018). The genotypes for the diploid calling were coded as *0* (*AA*), *1* (*AB*), or *2* (*BB*). For the tetraploid parameterization they were coded as *0* (*AAAA*), *1* (*AAAB*), *2* (*AABB*), *3* (*ABBB*), and *4* (*BBBB*). A third parameterization (assumption-free method) was used, which considered allele ratio #A/(#A+#a), where #A is the allele count (sequencing depth) of the alternative allele and #a is the allele count of the reference allele. No dosage calling was performed in this model (File S1); these data varied continuously between *0* and *1*.

### Population genetics analysis

In order to compare the information captured by each genomic-based relationship matrix, we performed linkage disequilibrium (LD), and principal components (PC) analyses. Pearson correlation tests (r^2^) were performed for pairwise LD estimation among SNPs within scaffolds, considering draft reference genomes (Bian *et al.* 2014; Gupta *et al.*, 2015). One SNP was randomly sampled per probe interval, and a total of 22,914 SNPs were used in the analysis. LD was obtained for all marker-based scenarios: i) diploid (*G2*); ii) tetraploid (*G4*) and iii) ratio (*i.e.*, continuous genotypes; *Gr*). The LD decay over physical distance was determined as the mean distance at the LD threshold of r^2^ = 0.2. To compare the LD among scenarios, the mean distances (Kb) and their interval confidences at r^2^ = 0.2 were compared. The diversity captured from each relationship matrix was obtained by PC using the R package adegenet v. 1.3-1 (Jombart and Ahmed 2011).

We also evaluated the observed heterozygosity in the population. For this, we obtained the ratio between the number of heterozygote genotypes and the total number of individuals. To estimate the heterozygosity for the continuous genotypes, empirical limits were established based on the mean and standard deviations presented for homozygotes classes of the tetraploid parameterization.

### Models

One-step single-trait Bayesian linear mixed models were used to predict breeding values for each individual in the population, as follows:

$$\bar{y}=\mu +Xb+Z_{1}c+Z_{2}r+Z_{3}a+Z_{4}bxa+e$$ (1)

Where $\bar{y}\;$is a vector of the phenotypic values of the trait being analyzed, μ is the population’s overall mean, *b* is the fixed effect of year, *c* is the random effect of *i*th column position in the field ∼ *N* (0, $I\sigma_{c}^{2}$), *r* is the random effect of the *i*th row position in the field ∼ *N* (0, $I\sigma_{r}^{2}$), *a* is the random effect of genotype ∼ *N* (0,$G_{a}\sigma_{a}^{2}$), where $G_{a}$ was replaced by the different additive relationship matrices as described in the next section. The *bxa* is the random effect of the year by genotype interaction ∼ *N* (0, $I\sigma_{bxa}^{2}$), and *e* is the random residual effect ∼ *N* (0, $I\sigma_{e}^{2}$). Row and column effects were considered nested within year only for the traits evaluated in two years. For traits measured in a single year, the same equation (1) was used without the year and the year by genotype interactions. The variance components for each random variable were: additive ($\sigma_{a}^{2}$), column ($\sigma_{c}^{2}$), row ($\sigma_{r}^{2}$), year-by-genotype interaction ($\sigma_{bxa}^{2}$), and residual ($\sigma_{e}^{2}$). $X,\;Z_{1},\;Z_{2},\;Z_{3},$ and $Z_{4}$ were incidence matrices for year, column, row, genotype, and year by genotype interaction, respectively. The narrow-sense heritabilities were estimated considering the ratio between the additive variance component and the total phenotypic variance (sum of all variance components).

### Relationship matrices

To quantify the effect of the genetic information used to build the relationship matrices on the predictive ability (PA), we performed analyses considering different approaches to modeling the genotypic values in autotetraploid species (Table 1, File S1). The factors tested were: i) the source of information used to build the relationship matrix (pedigree, genomic, or no relationship information); and ii) ploidy information (diploid, tetraploid, and assumption-free method).

**Table 1:** Methods and assumptions used to compare the influence of relationship matrices, ploidy and continuous genotypes in the prediction of breeding values for blueberry

Relationship matrix	Model	Ploidy assumption	Methodology
Identity	*I*	none	none
Pedigree-based	*A2*	2	Henderson (1976)
	*A4*	4	Kerr *et al.* (2012)
Marker-based	*G2*	2	VanRaden (2008)
	*G4*	4	
	*Gr*	none	

The methods chosen to obtain the relationship matrices are shown in the Table 1. The R package AGHmatrix v. 0.0.3003 (Amadeu *et al.* 2016) was used to obtain all relationship matrices (description of matrices File S1). The pedigree-based relationship matrices (*A*) were built considering a diploid model (Henderson 1976) and autotetraploid model without double-reduction (Kerr *et al.* 2012). The marker-based relationship matrices (*G*) were based on the incidence matrices of markers effects (*X*) according to VanRaden (2008) and adapted by Ashraf *et al.* (2016). Different assumptions can be made regarding the marker allele dosage in autotetraploids (Table 2). We built the *X* matrices under three assumptions regarding the additive marker allele dosage effect: *i)* a pseudo-diploid model, where all the heterozygous genotypes were assumed as one class, corresponding to a unique effect (data coded as *0*, *1*, and *2*); *ii)* an additive autotetraploid model, where each genotype had a specific value, and cumulative additive effect was assumed (data coded as *0*, *1*, *2*, *3*, and *4*); and *iii)* an assumption-free method based on the ratio of reads count for the alternative and reference alleles (continuous parameterization, assuming values between *0* and *1*), where also a cumulative additive effect was assumed. For the construction of the relationship matrices based on marker data, the missing genotypes were substituted by the mean.

**Table 2:** Theoretical genotype codes for marker-allele dosage effects considering pseudo-diploid, autotetraploid and continuous parameterizations. Adapted from Slater *et al.* (2016)

Genotype	Pseudo-Diploid	Autotetraploid	Continuous values*^a^*
*AAAA*	0	0	0 - 1
*AAAB*	1	1	
*AABB*	1	2	
*ABBB*	1	3	
*BBBB*	2	4	

a Continuous value with a ploidy assumption-free parameterization.

### Model implementation

The six models described above (Table 1) were fitted using the R package (R Core Team 2018) BGLR v. 1.0.5 (de los Campos and Pérez-Rodríguez 2016). Predictions were based on 30,000 iterations of the Gibbs sampler, in which 5,000 were taken as burn-in, and a thinning of five. The number of iterations, burn-in, and thinning interval parameters were evaluated to define the final values used in the analysis (Figure S1). A single step regression approach was applied to perform all phenotypic BLUP (I matrix), pedigree-BLUP (P-BLUP), and genomic-BLUP (G-BLUP). Default hyper-parameters were used, as previously described (Pérez and de los Campos 2014).

### Validation and model comparison

For each trait, models were compared based on their PA, stability (mean square errors), goodness-of-fit, and expected genetic gain. A 10-fold cross validation scheme was applied to compute model PA, for this the genotypes were assigned to ten groups, on each cross-validation step the phenotypic information for one of the groups was omitted (validation set) and predicted considering the model obtained from the remaining nine groups (training set). Because each validation group might have a different mean (Resende *et al.* 2012b), the phenotypic PA were obtained as the Pearson correlation coefficient between the empirical best linear unbiased estimation values (eBLUEs) obtained by considering all the variables in the equations 1 as fixed (*i.e.*, Least Square means estimations; LSMeans) and the cross-validated breeding values (BV) predicted by the models for each validation fold. The goodness-of-fit for the different models was evaluated with measures of DIC (Spiegelhalter *et al.* 2002) obtained from the full data set, extracted from the object returned by BGLR. The model with the lowest value for this parameter defined the best fit for the data. For the expected genetic gain estimation we used the following formula: ΔG = ($PA\cdot \sigma_{a}\cdot i$)/*L*, where *PA* is the phenotypic predictive ability, $\sigma_{a}$ is the square root of additive genetic variance in the population, *i* is the selection intensity, and *L* is the breeding cycle length. To make it comparable between methods the selection intensity (*i*) was considered constant for all methods and equal to 1.

Average phenotypic and raw genotypic data used during the current study will become available to promote further studies on the effect of dosage calling in the context of GS modeling.

### Data availability

Phenotypic datasets (eBLUES) are available from the Dyrad Digital Repository (accession number doi: 10.5061/dryad.kd4jq6h). Genotypic data and supplemental material are available at Figshare. Files include diploid, tetraploid and continuous genotypes, supplemental information 1 to 5, which includes: 1) description of the matrices used in the study; 2) model convergence figure; 3) LD distribution per parameterization; 4) principal components plots for each parameterization; 5) table of the predictive abilities, MSE, goodness-of-fit and beta for each parameterization. The authors ratify that all data necessary for confirming the conclusions of the article are present within the article, figures, and tables. Supplemental material available at Figshare: https://doi.org/10.25387/g3.7728365.

## Results

### Population genetics analyses

**Figure 1:**
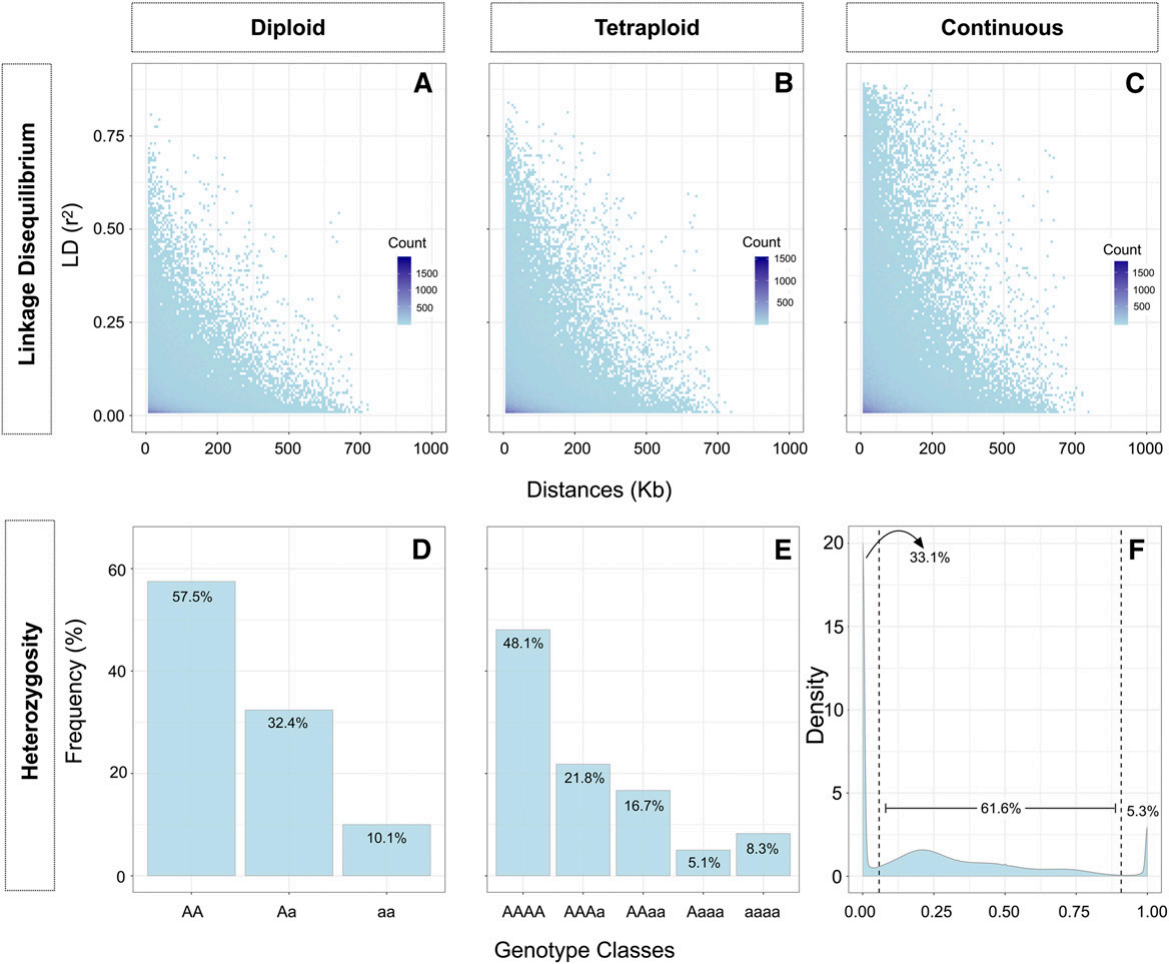
Linkage disequilibrium decay and heterozygosity for blueberry. Linkage disequilibrium decay estimation using one marker per probe, within scaffolds for (A) diploid, (B) tetraploid and (C) continuous genotype parameterizations. Heterozygosity observed in (D) diploid, (E) tetraploid, and (F) heterozygosity empirically established for the continuous genotypes’ scenario, assuming the limits of 0.058 ≤ X ≤ 0.908.

Linkage disequilibrium decayed below r^2^ = 0.2 at distances of 88.3 Kb, 92.6 Kb, and 98.2 Kb for the diploid, tetraploid and continuous models, respectively (Figure 1A-C). No significant difference was observed considering the confidence interval for the mean distance (Kb) at r^2^ = 0.2 among different ploidies and continuous genotyping scenarios (Figure S2).

Similarly, no major differences were found between parameterizations within methodology (*i.e.*, pedigree-based or marker-based methods) in the PC analysis (Figure S3). The first two PC components of the marker-based (*G*) matrices were consistent across all matrices, explaining approximately 20% of the variation, *G2* matrix captured 20.60% of the variation, while *G4* captured 21.71%, and *Gr* captured 23.36% (Figure S3 A-C). The PC analysis results were consistent between pedigree methodologies as well. Approximately 38% of the variation was explained (*i.e.*, 37.74% of the variability was explained for the *A2* matrix and 37.86% was explained for the *A4* matrix, Figure S3 D-E). The results obtained in the PC analysis did not justify a stratified sampling of cross-validation populations, since no evidence of sub-population structure was detected for any of the relationship matrices.

**Figure 2:**
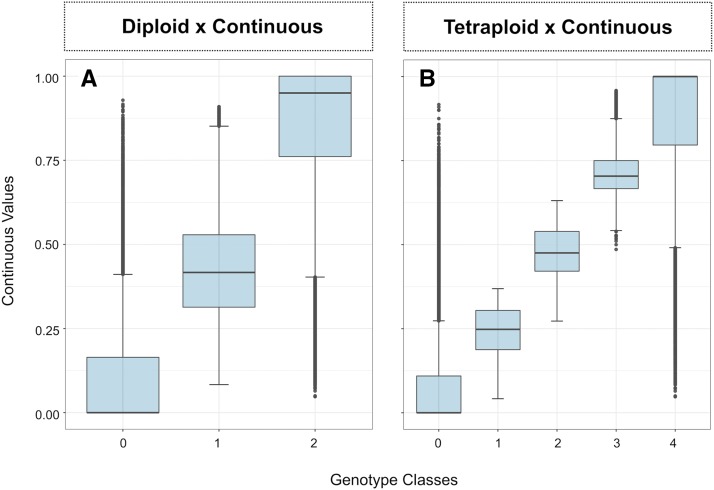
Relationship between continuous values and the classes assumed in the (A) diploid and (B) tetraploid parameterizations.

Considering the heterozygosity observed in each scenario, genotypes assumed as homozygotes in the diploid parameterization were classified as one of the possible heterozygote classes in the tetraploid and in the assumption-free parameterizations (Figure 1D-F). As a result of this process, the tetraploid parameterization presented 37.50% more heterozygotes than the diploid parameterization. Considering the empirical thresholds established to compare the proportion of “heterozygotes” in the continuous genotypes with the ploidy parameterizations, values equal to or below 0.058 and equal to or above 0.908 were considered as “homozygotes” classes (dashed lines, Figure 1F). With this, 61.59% of the genotypes were considered “heterozygotes”, thus the continuous method would have presented 89.92% and 41.23% more heterozygotes than the diploid and the tetraploid parameterization, respectively. Nevertheless, some misclassification of data into classes in the diploid and tetraploid parameterization might have occurred (Figure 2A-B).

### Variance estimates

The posterior means of the genetic parameters are summarized in Table 3. All the traits presented additive genetic variance significantly higher than zero. A wide range of variance was observed within a given parameter for the different methodologies, and most of the values were significantly different from each other (considering Tukey test results; Table 3, Table S1). Marker-based methodologies generated significantly smaller estimations for variance components when compared with pedigree-based estimations. Within marker-based methodologies, the assumption-free parameterization generated significantly smaller estimations. The effects of the difference in the estimation of variance components are reflected in the estimated heritabilities – smaller values were estimated for marker-based methodologies. The lowest heritability was obtained for soluble solids, flower buds, and pH. Considering all methods, narrow-sense heritability values varied between 0.152 and 0.574, for flower buds and fruit weight, respectively.

**Table 3:** Genetic parameters estimated for eight yield and fruit-related traits analyzed with six linear mixed models, considering the use of ploidy information and continuous genotypes. Source of information, and dosage parameterizations for the relationship matrices indicated by the letters (*I*, *A*, or *G*), and index 2, 4, and r respectively*

Trait	Relationship matrix	Additive Variance	Residual Variance	Heritability	EGG 2014^1^	EGG 2015^1^
Soluble Solid (°Brix)	*I*	0.806 b	1.794 d	0.257 a	0.018 b	—
	*A2*	0.777 c	2.129 b	0.239 b	0.021 ab	—
	*A4*	0.764 c	2.125 b	0.236 b	0.021 ab	—
	*G2*	0.848 a	2.026 c	0.262 a	0.028 a	—
	*G4*	0.673 d	2.109 b	0.215 c	0.026 a	—
	*Gr*	0.546 e	2.241 a	0.174 d	0.022 ab	—
Flower Buds	*I*	2.133 a	4.752 d	0.270 a	—	0.018 a
	*A2*	1.247 cd	6.080 a	0.153 de	—	0.019 a
	*A4*	1.232 d	6.070 a	0.152 e	—	0.018 a
	*G2*	2.106 a	5.562 c	0.251 b	—	0.030 a
	*G4*	1.526 b	5.881 b	0.188 c	—	0.025 a
	*Gr*	1.315 c	6.115 a	0.161 d	—	0.023 a
Fruit Diameter	*I*	2.236 f	6.804 b	0.162 f	0.047 b	0.041 c
	*A2*	3.647 a	6.854 b	0.250 a	0.063 b	0.054 bc
	*A4*	3.581 b	6.825 b	0.247 b	0.061 b	0.054 bc
	*G2*	3.428 c	6.799 b	0.242 c	0.088 a	0.079 a
	*G4*	2.992 d	6.954 ab	0.216 d	0.083 a	0.072 ab
	*Gr*	2.910 e	7.219 a	0.207 e	0.082 a	0.071 ab
Fruit Firmness	*I*	509.180 f	737.735 b	0.275 f	0.567 c	0.798 c
	*A2*	806.908 a	741.089 b	0.401 a	0.881 b	1.16 b
	*A4*	786.601 b	742.547 b	0.395 b	0.877 b	1.135 b
	*G2*	725.192 c	734.332 b	0.376 c	1.243 a	1.511 a
	*G4*	659.584 e	749.865 b	0.351 e	1.217 a	1.446 a
	*Gr*	687.685 d	783.729 a	0.354 d	1.257 a	1.490 a
pH	*I*	0.053 a	0.118 d	0.253 a	0.005 a	—
	*A2*	0.052 a	0.140 c	0.241 b	0.006 a	—
	*A4*	0.052 a	0.140 c	0.238 b	0.005 a	—
	*G2*	0.052 a	0.141 c	0.241 b	0.007 a	—
	*G4*	0.040 b	0.147 b	0.191 c	0.006 a	—
	*Gr*	0.035 c	0.153 a	0.165 d	0.006 a	—
Fruit Scar	*I*	0.086 f	0.073 d	0.381 f	0.008 c	0.009 c
	*A2*	0.139 a	0.075 c	0.528 a	0.013 b	0.014 b
	*A4*	0.135 b	0.075 bc	0.522 b	0.013 b	0.014 b
	*G2*	0.123 d	0.075 cd	0.500 c	0.018 a	0.018 a
	*G4*	0.115 e	0.077 b	0.479 e	0.018 a	0.017 a
	*Gr*	0.126 c	0.081 a	0.494 d	0.019 a	0.018 a
Fruit Weight	*I*	0.217 f	0.214 b	0.374 f	0.013 c	0.014 c
	*A2*	0.403 a	0.207 c	0.574 a	0.021 b	0.021 b
	*A4*	0.393 b	0.205 c	0.568 b	0.021 b	0.021 b
	*G2*	0.344 d	0.206 c	0.535 c	0.030 a	0.029 a
	*G4*	0.323 e	0.215 b	0.513 e	0.029 a	0.027 a
	*Gr*	0.352 c	0.231 a	0.522 d	0.030 a	0.028 a
Yield	*I*	0.326 f	0.444 bc	0.310 f	0.012 b	0.015 c
	*A2*	0.549 a	0.442 bc	0.447 a	0.019 a	0.022 b
	*A4*	0.536 b	0.442 bc	0.441 b	0.020 a	0.021 b
	*G2*	0.470 c	0.441 c	0.407 c	0.026 a	0.030 a
	*G4*	0.421 d	0.458 b	0.374 d	0.024 a	0.028 a
	*Gr*	0.411 e	0.493 a	0.356 e	0.023 a	0.027 a

* Letters based on Tukey test performed considering estimations obtained from 10 independent runs of the full models with BGLR (equation 1).

1 Expected Genetic Gain on trait scale.

### Effect of the genetic information to build the relationship matrices

**Figure 3:**
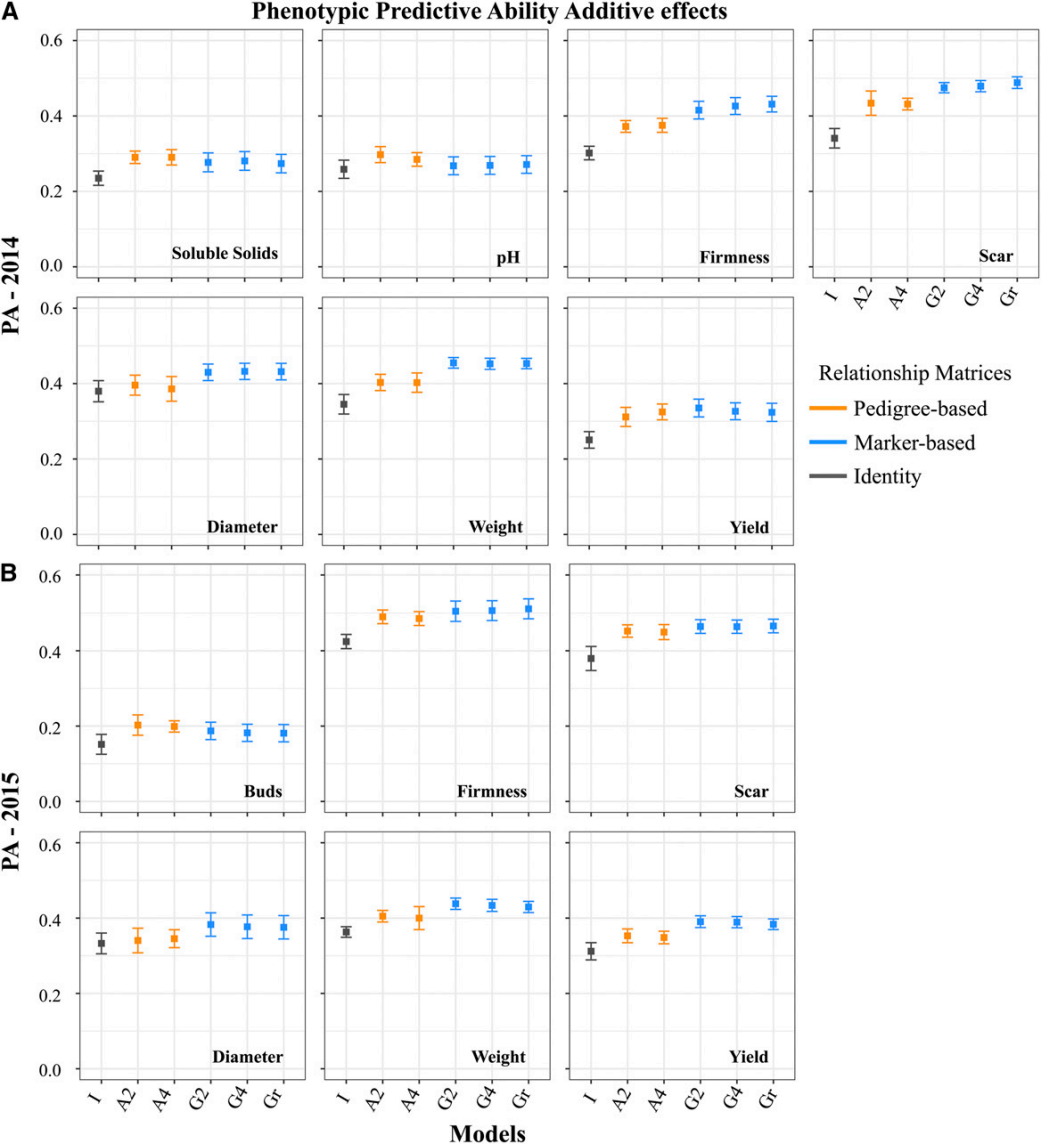
Phenotypic predictive abilities. Predictive abilities obtained for (A) seven traits in 2014, and (B) for six traits in 2015 considering different dosage parameterizations (indicated by the numbers *2* or *4*, and *r* for ratio values), and different relationship matrices (indicated by the letters *I*, *A*, and *G*) in the prediction of breeding values of 1,847 blueberry genotypes.

The incorporation of relationship information in the analysis generated better PA results than the phenotypic-BLUP model without it. Overall, we observed that higher values for the phenotypic PA were obtained when marker-based relationship matrices were used, when compared with phenotypic and pedigree BLUP (*I* and *A* matrices, respectively). However, the marker-based and pedigree-based results were not always significantly different from each other (Figure 3, Table S1). The use of molecular data yielded phenotypic PA values ranging from 0.27 (pH) to 0.49 (fruit scar) in 2014, and from 0.15 (flower buds) to 0.51 (fruit firmness) in 2015. Lower PA values were obtained for traits with lower heritability and better results were observed for the second year of evaluation. The biggest increase in the PA values can be seen for fruit firmness – when we compared marker and pedigree results, we observed an average increase of 13.37% in 2014. Also, an increase in the PA values of 11% was observed for fruit diameter and yield in 2015 when markers were used instead of pedigree data.

The use of pedigree-based relationship matrices generated higher phenotypic PA values for all the traits, when compared with the assumption of unrelated individuals (*i.e.*, identity matrix). Unlike the identity matrix, the use of pedigree-based matrix assumes that there is relationship (expected values) among individuals. The phenotypic PA obtained for the pedigree methods in 2014 yielded values from 0.20 (flower bud) to 0.49 (fruit firmness). As with marker-based methods, smaller values were observed for traits with lower heritability (*i.e.*, pH, brix, and flower bud). For 2015, the PA results for the phenotypic-BLUP were 0.36, 0.38, and 0.42, for fruit weight, fruit scar, and fruit firmness, respectively. The PA values obtained for the same traits with pedigree-BLUP were 0.40, 0.45, and 0.49, respectively. No significant differences between the models’ stability were observed (Table S1).

### Use of dosage information and continuous genotypes

Our results indicate that the importance of dosage in GS will vary depending on the trait being analyzed. For example, in 2014 the PA for fruit firmness, fruit scar, and fruit diameter showed modestly better phenotypic PA when the tetraploid and continuous parameterizations were applied, as opposed to the diploid parameterization (Figure 3, Table S1). The addition of more classes for the representation of the genotypic classes added complexity to the models (Table S1), in other words bigger values of DIC were observed for *G4* and *Gr* models. Although no significant difference was observed between marker-based models, the use of relationship matrices derived from continuous genotype data (ploidy-free parameterization) performed equally well as the best models (Figure 3, Table S1).

### Expected genetic gain in a perennial fruit tree, blueberry

**Figure 4:**
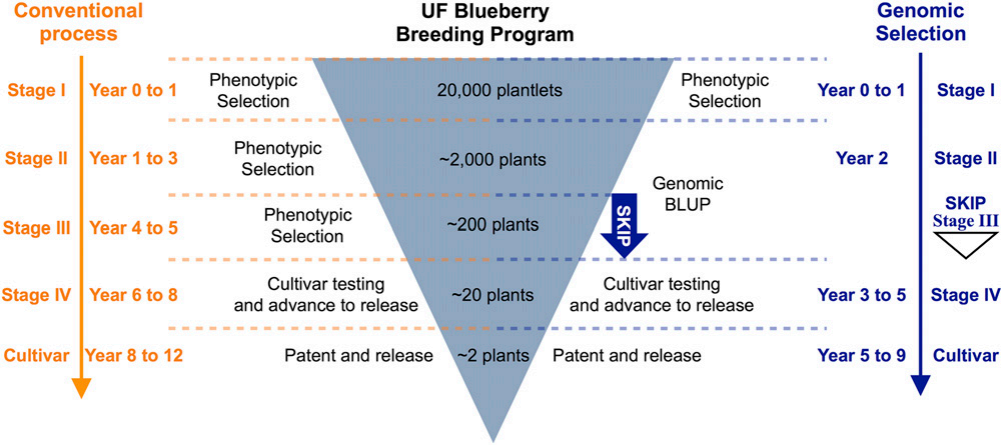
Proposal of GS implementation in the University of Florida blueberry breeding program. UF blueberry breeding program stages and times of selection considering the conventional process (left) compared with the proposed process implementing genomic selection (right).

The results obtained for the expected genetic gain (EGG) are summarized in Table 3. GS offers the possibility to accelerate genetic improvement by decreasing the breeding cycle and selecting superior individuals earlier in the breeding program. Considering a breeding cycle (*L*) of 12 years (Cellon *et al.* 2018) we propose that routine genomic selection could be implemented in the second stage of the blueberry breeding program, which would allow the omission of a whole stage (stage III), and a three-year reduction for cultivar release (Figure 4).

Higher EGG was obtained for all traits when marker-based matrices (*i.e.*, genomic selection) were applied (Table 3), which was mainly related to the reduction in cycle time. The implementation of GS in the second stage population would lead to an increase in the EGG varying from 27% (pH) to 119% (scar) when compared with the application of phenotypic BLUP. Considering the comparison of marker-based and pedigree-based models, an increase of 15% (pH) to 41% (fruit weight, fruit scar, and flower buds) in the EGG was observed (Table 3). In addition, the use of continuous data generated EGG values that were not significantly different of the best models for all traits (Table 3).

## Discussion

In this study, six approaches were applied to predict breeding values for eight yield and fruit-quality traits measured in a real blueberry breeding population. Analyses were based on phenotypic, pedigree, and high-density marker data from 1,847 individuals. We compared the expected genetic gain, the stability, and the PA of models considering different sources to build the relationship matrices (only phenotype = BLUP, phenotypes + pedigree = P-BLUP, phenotypes + genomic = G-BLUP). Our results also explored models accounting for ploidy information and compared the use of genotypic data that is independent of assumptions regarding ploidy levels (continuous) to perform GS, avoiding the need for *a priori* parameterization for a given ploidy level.

### Continuous data

Our research showed empirical evidences that the use of continuous genotypic data from NGS can be effectively applied in GS models for autotetraploid species. This method was tested and compared with marker calling methodologies at the individual level in genome wide association studies (Grandke *et al.* 2016). It was also tested in family pool data for GS (Ashraf *et al.* 2014; Cericola *et al.* 2018; Guo *et al.* 2018), as well as used at the individual level in tetraploid potato for GS by Sverrisdóttir *et al.* (2017). However, to our knowledge the comparison of continuous genotypes with ploidy parameterizations for genomic selection has not yet been reported. Here we empirically compare diploid, tetraploid, and continuous data at the individual level for the application of genomic selection in an autotetraploid species.

In polyploids, the assignment of genotypic classes based on NGS data has been a major challenge, with high risk of misclassification (Grandke *et al.* 2016, Bourke *et al.* 2018). The problem is further exacerbated as the ploidy increases – for a given level of ploidy, *n*, the expected number of genotypic classes is *2n+1*. As a consequence, the signal distribution derived from each genotypic class increasingly approximates a continuous distribution, where no clear separation is observed (Grandke *et al.* 2016). Despite extensive research to address these challenges (Serang *et al.* 2012), advances have been mostly limited to SNP arrays in tetraploid data (Schmitz Carley *et al.* 2017). Studies that evaluated genotype calling with NGS data obtained from polyploids show that no method works properly, and that misclassification of genotypes can significantly interfere in the results of genetic studies (Grandke *et al.* 2016). This misclassification can be observed in our results when are diploid, or tetraploid parameterization were used in the genomic data (Figure 2A-B), even with our high sequencing depth and with standard parameters of filtering. The use of the continuous genotyping approach provides a relevant alternative to overcome this issue that is independent of assumptions regarding ploidy level. Models that used continuous genotypic data performed as well as the best models and resulted in modestly better predictive abilities for some of the traits (*i.e.*, fruit firmness, fruit scar, and fruit diameter; Table 3), which could indicate better prediction of future populations. The use of continuous genotypes also simplifies the analysis complexity and time, by eliminating the genotype calling and parameterization for a give ploidy, because instead, the ratio of reads assigned to each allele are used. The benefits of continuous genotyping could easily be extended to more complex polyploids (higher ploidies), where the genotype attribution is even more difficult, however higher sequencing depth would be required. Meanwhile, for more complex models, such as those that consider dominance effects, dosage calling is still necessary.

### Relationship matrices

Our results also showed that including information based on the genetic merit of the individuals yielded better results when compared with the phenotypic-BLUP analysis (based on the identity matrix; Table 3), corroborating previous studies in the literature (*e.g.*, Muir 2007; Resende *et al.* 2012a; Muñoz *et al.* 2014a). In addition, the use of marker-based methodologies generated better predictions than pedigree for most of the traits. Marker-based methods allow the capture of Mendelian segregation (Daetwyler *et al.* 2013;). This is especially important in our population, since it was composed of 117 full-sib families. In this context, pedigree-based methods have no power to distinguish variance within families. Another advantage is that marker-based methods allows the computation of genetic similarity among unidentified individuals in the pedigree, and corrections of errors in the pedigree, which can affect parameter estimation causing reduction in the genetic gain (Muñoz *et al.* 2014b).

In our results, some non-significant differences between pedigree and marker-based methods were identified, which could be an effect of the extensive pedigree data used, as well as bias in pedigree-based estimations. Pedigree-based methods can overestimate the reliability of selection and consequently, the accuracy (Bulmer 1971; Gorjanc *et al.* 2015). Furthermore, it also presents low efficiency to capture and estimate genetic relationships among individuals (Resende *et al.* 2017).

It is interesting to notice that we used extensive pedigree information that dates back to 1907 for our predictions, which may not be common in other autopolyploid breeding. This extensive information can have significant implications on the estimation of relationship coefficients (Amadeu *et al.* 2016) and consequently, in breeding value predictions. Therefore, for breeding programs with smaller pedigree depth information, the comparison between accuracies of prediction from marker and pedigree-based methodologies could be even bigger than what was found in our study.

### Allele dosage

The results obtained for both models that assumed more than three genotypic classes (*G4* and *Gr*) demonstrate the importance of considering dosage in the prediction of breeding values. However, this will depend on the trait analyzed, as previously reported by Nyine *et al.* (2018) and Endelman *et al.* (2018). For example, modest improvement was verified in the PA for fruit firmness, fruit scar, and fruit diameter when this factor was considered in the models. The addition of classes for the representation of ploidy increased the complexity of the models (Figure 3, Table 3, Table S1) however, these assumptions also show a more realistic representation of the nature of the species. The inclusion of nonadditive effects into the models could also improve model accuracy. Endelman *et al.* (2018) demonstrated that the inclusion of digenic effects, as well as accounting for ploidy information, presented a higher accuracy over diploid models when using a SNP array.

### Genomic selection for perennial autopolyploids

We also demonstrate the value of applying GS in a perennial fruit tree, blueberry. One cycle of blueberry breeding takes from 12 to 15 years until the release of a new cultivar (Lyrene 2008; Cellon *et al.* 2018). By applying selection based on high-density markers at early stages of the program, the time to cultivar release could decrease by three years (Figure 4), significantly improving the expected genetic gain per unit of time. More specifically, the use of GS would lead to an average increase of 86% in the EGG when compared with phenotypic BLUP, and an average increase of 32% over the application of pedigree-based models (Table 3). Implementing GS as we propose here could eliminate one stage in the breeding and selection process toward cultivar development, which will reduce costs associated with field trials and phenotyping. The implementation of GS would require extra financial outlay when genotyping and accurately phenotyping the training population. However, the savings on phenotyping and field trials of future generations (selection populations) could result in a break-even financial exercise, and as a result could be a cost-effective application of GS. However, this financial analysis needs to be performed for each crop in a case-by-case basis. To promote further studies on the effect of dosage calling using NGS, as methods and software improve, we are providing genotypic and phenotypic data to use as comparison of methods in the context of GS.
